# Nano‐Phase Separation and Analyte Binding in Aptasensors Investigated by Nano‐IR Spectroscopy

**DOI:** 10.1002/smll.202409369

**Published:** 2025-01-19

**Authors:** Nafiseh Samiseresht, Gabriela Figueroa Miranda, Ankita Das, Kevin Graef, Dirk Mayer, Martin Rabe

**Affiliations:** ^1^ Department of Interface Chemistry and Surface Engineering Max Planck Institute for Sustainable Materials 40237 Düsseldorf Germany; ^2^ Institute of Biological Information Processing Bioelectronics (IBI‐3) Forschungszentrum Jülich GmbH 52428 Jülich Germany

**Keywords:** aptasensor, nano‐IR spectroscopy, nano‐phase separation, self‐assembled‐monolayer, single molecule spectroscopy

## Abstract

Biosensors based on DNA aptamer receptors are increasingly used in diagnostic applications. To improve the sensitivity and specificity of aptasensors, parameters affecting the stability and binding efficiency of the receptor layer need to be identified and studied. For example, the blocking step, i.e., the addition of inert molecules to the receptor layer, can improve sensor performance, but can also cause phase separation into nanodomains of unknown composition and structure. Here, nano‐IR spectroscopy is used together with complementary macroscopic spectroscopic methods to study the nano‐structural variations during the fabrication of a recently developed SARS‐CoV‐2 aptasensor. The blocking step by polyethylene glycol (PEG) causes a significant thickening of the receptor layer and a phase separation into nanodomains consisting of an aptamer‐rich and a slightly thicker PEG‐rich phase. The unambiguous chemical identification of the nanodomains is achieved by analysis of nano‐IR images. Furthermore, bound analyte (spike protein of SARS‐CoV‐2) is detected at the single molecule level. Detailed analysis of the local nano‐IR spectra revealed structural properties such as the amorphous state of the PEG molecules within the nanodomains and a strong change in the secondary structure of the analyte. This study significantly advances the understanding of nanoscale chemical processes in the receptor layer of aptasensors.

## Graphical abstract

An atomic force microscopy ‐ infrared spectroscopy (AFM‐IR) study on the fabrication of an aptamer‐based biosensor revealed the formation of nanodomains rich in DNA aptamers and blocking polyethylene glycol (PEG) molecules. Single‐molecule spectroscopy of the analyte protein is also reported. The local nano‐IR spectra revealed structural details such as the amorphous structure of the blocking agent and a secondary structure change of the analyte.

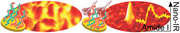

## 1 Introduction

Biosensors based on self‐assembled monolayers (SAMs) of oligonucleotide aptamers have been developed in recent years as an alternative approach to antibody‐based biosensors and are able to selectively target analytes with high specificity and reusability.^[^ ^1^, ^2^, ^3^ ^]^ Aptamers are in most cases composed of random sequences of nucleotides obtained to bind a specific analyte via the systematic evolution of ligands by exponential enrichment (SELEX).^[^ ^4^, ^5^ ^]^ Because of several advantages over proteins such as longer shelf life, low batch to batch variation etc. aptasensors have the potential to overcome the shortcomings of biosensors based on enzymes or antibodies.^[^ ^6^, ^7^, ^8^ ^]^

For the application in biosensors, rational truncation of an aptamer has been shown to reduce the steric hindrance for the charge transfer involved in electrochemical sensing and increases the space for the analyte to bind. Hence truncated, shorter sequences are preferred for the design of electrochemical biosensing devices.^[^ ^5^, ^7^, ^9^, ^10^, ^11^ ^]^ For example, truncated C9t single‐stranded DNA (ss‐DNA) aptamer was employed in a recently developed biosensor to detect the spike glycoprotein (S protein) of the SARS‐CoV‐2 Omicron variant.^[^ ^12^, ^13^ ^]^ For fabrication of a gold based aptasensor, the aptamers which act as receptors are typically chemisorbed to the interface by Au–S bonds. Factors such as the stability of the receptors, their accessibility for the analyte, and the number of receptors per unit area are important for the sensor performance and the efficiency in detecting small analyte concentrations. Several strategies exist to enhance these factors. For instance, the stability of the receptor layer, particularly the binding of the aptamer to the surface, can be improved by using multi‐thiol aptamers.^[^ ^14^ ^]^ The intermolecular electrostatic repulsion between the DNA strands is minimized during self‐assembly by using a buffer with higher ionic strength, and the use of bivalent cations (such as Mg^2 +^), which results in higher coverage of aptamers on the surface due to screening of the DNA backbone charges.^[^ ^15^, ^16^ ^]^

Another commonly used method to improve aptasensor performance in several aspects is the incorporation of a blocking agent during the sensor fabrication.The blocking agent is a second thiol compound with an antifouling character which may be introduced in a mixed solution with aptamers or separately on an aptamer‐modified surface.^[^ ^15^, ^17^, ^18^, ^19^, ^20^ ^]^ Common blocking agents are thiolated alcohols or polyethylene glycols (PEGs), of which the latter are long known for their high resistance to protein and cell adsorption and are therefore well suited for complex biological environments.^[^ ^18^, ^21^, ^22^, ^23^, ^24^, ^25^, ^26^ ^]^ The molecules block the area, which remained uncovered by aptamers, and prevent nonspecific interaction of the analyte and other matrix components with the metal surface during detection. Next to this function, the blocking can influence the sensor functionality positively or negatively via several mechanisms: loosely adsorbed aptamers attached by nonspecifc interactions of nucleotides with the interface, are replaced by chemisorbed aptamers.^[^ ^16^, ^18^, ^19^, ^27^ ^]^ The same studies report the competitive replacement of chemisorbed ss‐DNA upon longer incubation times, which ultimately reduced the amount of ss‐DNA on the surface. Also, it was observed that ss‐DNA chains reorient to a more ordered and more vertical orientation, during the early stages of the blocking, which enhances DNA‐hybridization and hence detection efficiency.^[^ ^15^, ^16^, ^19^, ^27^, ^28^, ^29^, ^30^, ^31^ ^]^ It should be noted that many of these mechanistic studies investigated the influence of the blocking on the target binding in terms of DNA hybridization. However, distinct differences in the effect of the blocking agent on sensing might occur when the actual target is a protein since aptamer‐protein binding often requires a specific folding of the aptamer that exposes its binding sites.^[^ ^32^ ^]^ For instance, an increase in hybridization was reported when ss‐DNA was assembled in nano‐domains with high local density,^[^ ^33^ ^]^ while a reduced protein target binding was observed in phase separated aptamer/PEG films.^[^ ^17^ ^]^ Thus, detailed studies of the structure and morphologies of ss‐DNA/blocking agent mixed SAMs at the nanoscale is required for a knowledge‐based choice and improvement of blocking agents during aptasensor fabrication.

To obtain chemical information on the nanoscale, the photothermal AFM‐IR technique combines atomic force microscopy (AFM) with infrared (IR) spectroscopy, allowing for nanospectroscopy with resolutions down to 25 nm.^[^ ^34^ ^]^ AFM‐IR in resonance enhanced mode is monolayer sensitive and opens up great opportunities for the study of biomolecular systems.^[^ ^35^, ^36^, ^37^, ^38^ ^]^ Here, AFM‐IR in combination with classical IR reflection absorption spectroscopy (IRRAS) and spectroscopic ellipsometry (SE) is used, to study the individual fabrication steps of a recently developed electrochemical C9t aptasensor for the detection of S protein of the SARS‐CoV‐2 Omicron variant.^[^ ^13^ ^]^ To that end, aptamer binding and PEG blocking as well as the sensing of the S protein is examined. Following the morphological changes that occur in the respective steps allows conclusions on the molecular changes that happen. Chemical imaging as well as nano‐IR spectroscopic results give insight on the nature of different domains found in this phase‐separated system. Finally, the AFM‐IR method is used to examine binding events of the analyte to the receptor layer.

## 2 Results and Discussion

**Figure 1:**
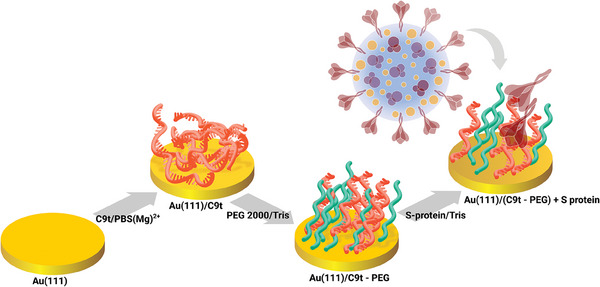
Modification steps during sensor fabrication and application. I: aptamer immobilization, II: PEG‐blocking, III: S protein binding.

The sensor surface was characterized after each subsequent preparation step (**Figure** **1**) which are I: aptamer immobilization, and II: PEG‐blocking. Also, the sensor was characterized after step III: S protein binding. Characterization was done by IRRAS, SE, and AFM‐IR. For IRRAS and SE measurements, hydrogen flame annealed thin films of Au(111) on glass were used as substrates, while in AFM‐IR Au(111) single crystals (sc) were employed.

### 2.1 Changes in Sensor Topography and Thickness During Fabrication

**Figure 2:**
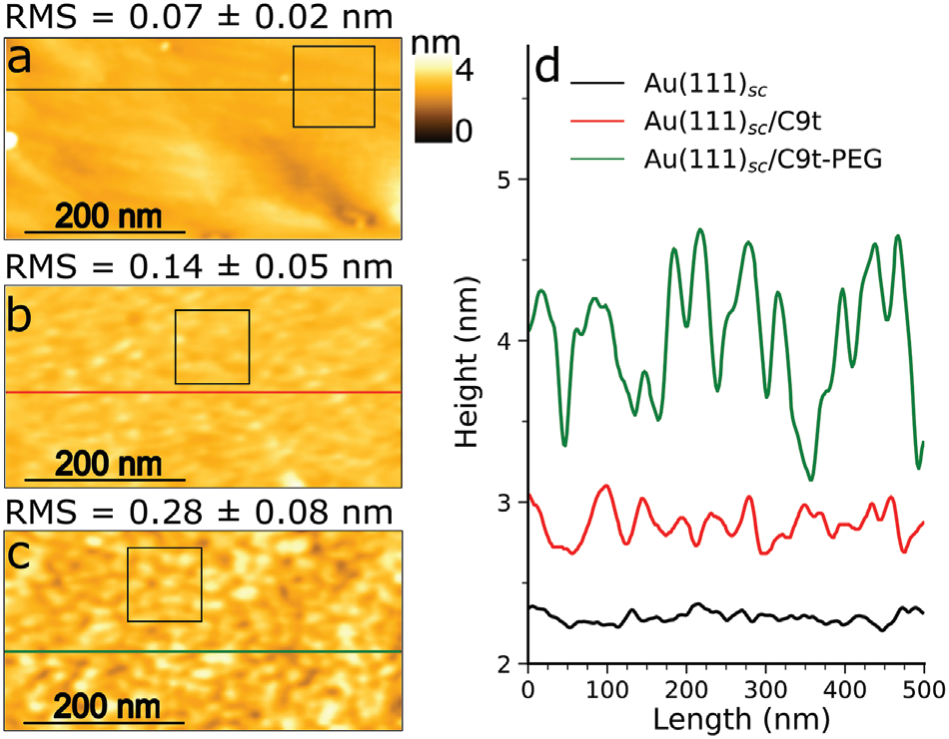
Results of TM AFM measurements during biosensor fabrication. Typical topographies of Au(111)_sc_ (a), Au(111)_sc_/C9t (b), and Au(111)_sc_/PEG‐C9t (c) and the respective line scans (d). 50×50‐pixel squares in the topography images indicate selected areas for roughness determination.

For each sensor fabrication step, the samples were characterized by tapping mode AFM‐IR. With this method, topographic and spectral information are obtained simultaneously, although in the following, the topography is presented first (**Figure** **2**). It is noted that granular features appeared in AFM topography at all sample conditions that are considered as substrate related features that do not interfere with the SAM formation (Discussion S1, Figures S1– S3, Supporting Information). Compared to the single crystalline Au(111)_sc_ interface, Au(111)_sc_/C9t showed a slightly roughened surface after aptamer immobilization. The pure Au(111)_sc_ interface shows atomically flat terraces that have a shallow root mean square (RMS) roughness value of 0.07 nm (Figure 2a). In comparison, the RMS roughness in a selected region on Au(111)_sc_/C9t is increased, but still relatively low with a value of 0.14 nm (Figure 2b). Line scans reveal typical height variations in the topography of Au(111)_sc_/C9t. While on the bare Au(111)_sc_ substrate mono‐atomic steps of approx. 0.25nm between terraces were found (Figure S1c, Supporting Information), Au(111)_sc_/C9t exhibits height variations up to 0.5nm (Figure 2d). The substrate employed here for the immobilization of C9t aptamer is atomically flat, hence the increase in roughness can be attributed to the formed DNA monolayer. The observed height differences on Au(111)/C9t are in the range expected for the diameter of an ss‐DNA chain which is estimated to be between 0.5 and 0.7 nm.^[^ ^39^, ^40^ ^]^ The second fabrication step, i.e., the incubation with PEG‐thiol, significantly altered the topography of the sensor interface. The AFM height image of Au(111)_sc_/PEG‐C9t exhibits nano‐domains and a further increased RMS roughness of 0.28nm (Figure 2c). The nano‐domains show typical height variations of approximately 1.0–1.5 nm as illustrated in the line profile (Figure 2d). The lateral size of the elevated domains was about 50nm.

**Figure 3:**
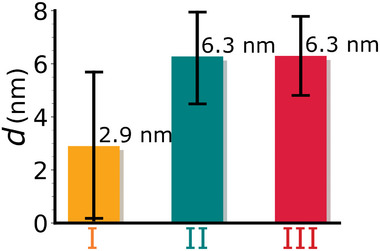
Layer thicknesses of I: Au(111)/C9t, II: Au(111)/PEG‐C9t, and III: Au(111)/(PEG‐C9t)+ S protein determined by spectroscopic ellipsometry. The error bars represent the maximum errors arising from the standard deviations of substrate and sample measurements.

Spectroscopic ellipsometry was used to determine the thicknesses of the SAMs on Au(111). To that end, the averaged ellipsometric angles Δ(λ) were fitted with an optical 3‐layer model that yielded the SAM thicknesses of Au(111)/C9t, Au(111)/PEG‐C9t, and Au(111)/(PEG‐C9t)+ S protein. The average SAM thickness (*d*) derived from the analysis of spectra acquired on Au(111)/C9t is 2.9 nm (**Figure** **3**). Samples of Au(111)/C9t feature a relatively high sample to sample variation in *d* (see also variations in Δ in Figure S5, Supporting Information), implying that it is difficult to control this parameter during the fabrication. This variation together with a relatively strong variation of the substrate's optical properties used for these measurements leads to a high relative error in the determined absolute thickness (Figure 3; Discussion S2, Supporting Information). Nevertheless, the average thickness is low compared to the molecular length of C9t. As a fully extended chain, the contour length of C9t would be about 27 nm.^[^ ^41^ ^]^ A more realistic worm‐like chain model of ss‐DNA (of comparable number of nucleotides) tethered to a conducting surface predicts an end‐to‐end distance in the range of 1015 nm.^[^ ^39^ ^]^ This estimation implies that the polymer‐like ss‐DNA chains must spread out on the surface to take in a flat conformation in Au(111)/C9t. This result is in common with the measured surface density (after blocking with MCH) that was determined to be about 0.013molecules nm^−2^,^[^ ^13^ ^]^ although before blocking this value might be up to an order of magnitude higher.^[^ ^40^ ^]^ Hence, the ss‐DNA chains lie flat on the surface and occupy the unblocked surface area, resulting in a relatively flat, but as shown by AFM, homogenous layer.

The average SAM thickness was found to increase upon PEG incubation to approx. 6.3 nm on Au(111)_sc_/PEG‐C9t (Figure 3), also the range of variation in *d* was reduced. It is noted that the absolute thickness increase may be slightly smaller considering that the true value and change of the film refractive index *n* (assumed here to be *n* = 1.43) cannot be known exactly in the studied system and that film thickness *d* and refractive index *n* are strongly correlated here (cf. Discussion S2, Supporting Information). Due to this correlation, the SE data might also be interpreted in terms of an increase in *n* due to a densification of the layer coinciding with the increase in *d*, as for instance observed upon blocking of ss‐DNA film with short alkanethiols.^[^ ^30^, ^31^ ^]^ An analysis of the ellipsometric raw data showed that the change in Δ and hence the increase in optical thickness (*n* × *d*) is statistically significant (Discussion S2, Supporting Information). Assuming a strong increase of *n* from 1.36 to 1.5 upon PEG blocking would result in a lower thickening from 3.3 nm on Au(111)/C9t to 5.7 nm on Au(111)/PEG‐C9t, which is still significant. Hence, the ellipsometric data clearly indicates that the blocking step induced a significant rise of the surface attached molecules while AFM indicates separation into phases with different heights. The average layer thickness still is considerably below the estimated end‐to‐end lengths of both C9t (1015 nm) and crystalline, i.e., helical PEG2000 (approx. 12 nm).^[^ ^42^ ^]^ Hence, although the observed thickness increase is interpreted in terms of an uprising of the ss‐DNA chains, the ss‐DNA molecules are most likely still in a coil conformational state on the sensor interface. The difference of the layer thickness with respect to the expected length of helical PEG might be caused by either a significant tilt of helical PEG chains or an amorphous conformation. Only, spectroscopic information can give clarity on this issue.

### 2.2 IRRA Spectroscopy Confirms Mixed Layer Formation

**Figure 4:**
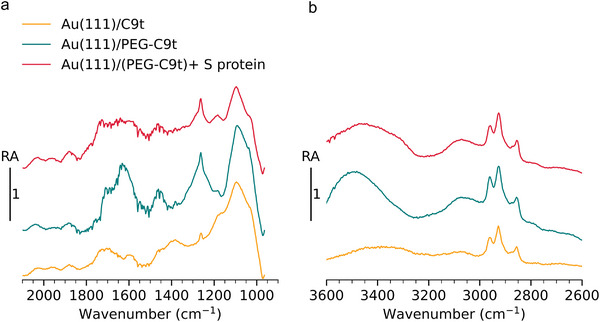
IRRA spectra of fabrication steps and application of aptasensor in the low (a) and high wavenumber range (b). Peak assignments are presented in **Table** **1** and in the text.

IRRA spectra recorded after each preparation step show peaks as expected for the molecules immobilized on the sensor surface (**Figure** **4**). The peak ratio varies from sample to sample which might be due to slight sample to sample variations in fabrication (Figure S6 steps I and II, Supporting Information) or variations in the substrate reflectivities (cf. Discussion S2, Supporting Information). The most prominent features confirming the presence of aptamer on the surface are two bands with maxima around 1080 cm^−1^ and 1260 cm^−1^, which appear in the spectra of all samples and are assigned to symmetric and asymmetric stretching vibrations (ν_*s*_, ν_*as*_) of (PO_2_)^−^ of the DNA backbone (**Table** **1**, Figure 4). The IR spectra of macromolecules such as DNA are featured by broad and complex bands that may be used as indicators for specific molecular properties. For instance, in transmission IR spectroscopy it was found that the hydration of DNA influences the shape of 1080 cm^−1^ and its shoulder at 1050 cm^−1^ assigned to the ν(C–O) of the sugar backbone.^[^ ^43^ ^]^ On the other hand the ν_*as*_(PO_2_)^−^ band position is known to be sensitive to both DNA conformation and hydration.^[^ ^44^, ^45^, ^46^ ^]^ Next to these molecular properties band intensities in IRRA spectra are very sensitive to molecular orientation and order of the examined moieties due to the so‐called selection rules on metal interfaces.^[^ ^47^ ^]^

**Table 1:** Band assignments and positions of absorbance bands (in cm^−1^) measured by AFM‐IR and IRRAS. Abbreviations: n.m.: not measured, NB: nucleobase, DB: deoxyribose (sugar).

	Au(111)_sc_/C9t	Au(111)_sc_/PEG‐C9t (aptamer‐rich)	Au(111)_sc_/PEG‐C9t (PEG‐rich)	Au(111)_sc_/(PEG‐C9t)+ S protein	Assignments vibration; molecule
AFM‐IR	1020−1050	1000−1050	1030−1050	1020−1080	backbone ν(C–O), ν_*s*_(PO_2_)^−^; DNA^[^ ^44^ ^]^
			1140		ν(C–O–C); PEG^[^ ^23^, ^58^ ^]^
	1205	1210	1210	1210	ν_*as*_(PO_2_)^−^; DNA^[^ ^44^ ^]^
	1460, 1580, 1720	1680 (very broad)			NB ν(C=O), ν(C=C), ν(C=N), ring vibrations; DNA^[^ ^44^ ^]^
				1550	Amide II; protein^[^ ^59^ ^]^
				1665	Amide I; protein^[^ ^59^ ^]^
	n.m.	2820, 2970	2860, 2930	2830, 2950	ν_*s*, *as*_(CH_2_; CH_3_)^[^ ^55^, ^56^, ^57^, ^60^ ^]^
	n.m.	3050	3050 (very weak)	3050 (very weak)	ν(C_*ar*_‐H); mostly NB in DNA^[^ ^60^ ^]^
	n.m.	3250‐3550	3250‐3550 (very weak)	3220‐3550	ν(NH), ν(OH), Amide A^[^ ^59^, ^60^ ^]^
	Au(111)/C9t	Au(111)/PEG‐C9t		Au(111)/(PEG‐C9t)+ S protein	assignments
IRRAS	1050	1050		1050	ν_*as*_(C–O); DB in DNA^[^ ^44^ ^]^
	1080	1080		1080	ν_*s*_(PO_2_)^−^; DNA^[^ ^44^ ^]^
	1260	1265		1265	ν_*as*_(PO_2_)^−^; DNA^[^ ^44^ ^]^
	1320‐1800	1465, 1630, 1710		1320‐1800	NB ν(C=O), ν(C=C), ν(C=N), ring vibrations; DNA^[^ ^44^ ^]^
	2860, 2930, 2960	2860, 2930, 2960		2860, 2930, 2960	ν_*s*, *as*_(CH_2_; CH_3_) ^[^ ^55^, ^56^, ^57^, ^60^ ^]^
	3075	3075		3075	ν(C_*ar*_‐H)

Additionally, the IR spectra of the molecules used here significantly overlap. Hence, complex variations in the IR spectra are observed with each fabrication step, which makes it difficult to assign unequivocally to specific effects. After PEG blocking on Au(111)/PEG‐C9t the ethyleneglycol C–O–C mode expected in the 1110 to 1140 cm^−1^ region, overlaps with the asymmetric ν_*as*_(PO_2_)^−^ and hence cannot be clearly identified. An enhanced intensity and broadening of the ν_*as*_(PO_2_)^−^ at 1265 cm^−1^ might imply significant reorientation of C9t after the blocking step. A series of broad bands from 1320 to 1800 cm^−1^ appear on Au(111)/C9t with lower intensity and are assigned to the ν(C═C) ring vibrations of the C9t nucleobases (thymin, guanine, adenine, cytosine) as well as ν(C═O and C═N). On Au(111)/PEG‐C9t these bands appear much stronger with main peaks at 1465 cm^−1^, 1630 cm^−1^, 1710 cm^−1^. At 1465 cm^−1^ PEG's δ(CH_2_) (scisorring) vibration may overlap with the DNA band. However, PEG molecules have no absorption above 1500 cm^−1^. Therefore, the higher intensities at 1630 cm^−1^ and 1710 cm^−1^ might be caused by a significant change in the orientation of the C9t chains. On Au(111)/(PEG‐C9t)+ S protein broad bands appear in this region which come from an overlap of several vibrations including aptamer rings, and protein amide I and II on the surface (Table 1).

In the higher frequency region from 2700 to 3600 cm^−1^, the ν_*s*_(CH_2_) at 2860 cm^−1^, ν_*as*_(CH_2_) at 2930 cm^−1^, ν_*as*_(CH_3_) at 2960 cm^−1^ on Au(111)/C9t, Au(111)/PEG‐C9t and Au(111)/(PEG‐C9t)+ S protein are observed since DNA, PEG as well as proteins contain aliphatic chains. A very mild broad absorption around 3075 cm^−1^ is assigned to unsaturated CH stretching vibrations ν(C_*ar*_‐H) that appears stronger on Au(111)/PEG‐C9t and Au(111)/(PEG‐C9t)+ S protein. Overall the IRRAS spectra indicate that the ss‐DNA is attached to the gold interface and that the PEG blocking does have a strong influence on the sample structure. However, a spectroscopic examination of the nano‐scale domains is not possible with this macroscopic method. Thus in the next step, the sensor fabrication is studied by nano‐IR spectroscopy.

### 2.3 Nano‐IR Spectroscopy Reveals Chemical Composition of the Nano Domains

**Figure 5:**
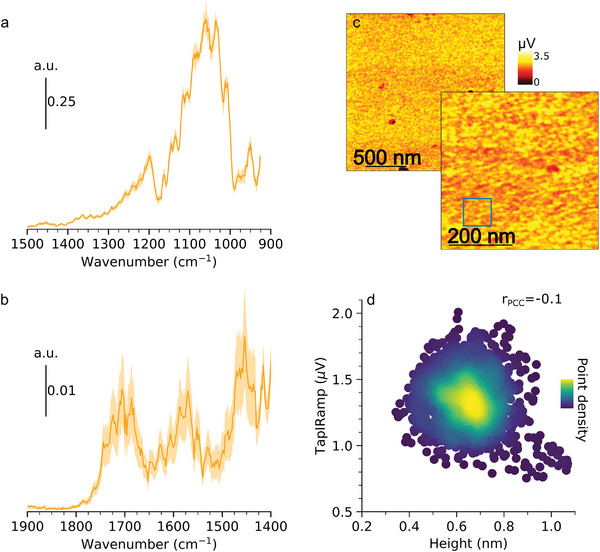
Results of TM AFM‐IR measurements on Au(111)_sc_/C9t. Averaged nano‐IR point spectra, recorded at 5 positions (a,b) (The spectra before correction and the position of recording are given in Figure S4, Supporting Information). Nano‐IR mappings at maximum of ν_*s*_(C═O) at 1720 cm^−1^ (c) (Associated topography and phase images are given in Figure S4, Supporting Information). IR absorbance‐height scatter plot with point density represented by the color (d) created from the data in the square indicated in (c).

Nano‐IR spectra of Au(111)_sc_/C9t in the spectral range considered here have four main regions with broad peaks. In the region from 1000 to 1430 cm^−1^, a broad absorption spans in the range between 1000 to 1150 cm^−1^, maximum absorbance around 1050 cm^−1^ (**Figure** **5**a). As represented in Table 1, contribution to this band arises mainly from DNA backbone vibrations, i.e., the ν(C–O) of deoxyribose and the symmetric stretching of the phosphodiester, ν_*s*_(PO_2_ ${\mathrm{PO}}_{2}^{-}$)^−^.

A medium intense peak at 1205 cm^−1^ is assigned to asymmetric ν_*as*_(PO_2_)^−^. It is noted that peak intensities are not comparable quantitatively over the full spectral range because the spectral range is covered using four different QCL stages which are tuned independently. Due to the independent tuning the transition steps of the lasers featured sharp intensity steps (Figure S4, Supporting Information) which were corrected for the spectra presented here. This might cause lower peak intensities in the higher frequency regions with respect to the lower frequency peaks. In the region from 1400 to 1800 cm^−1^, a series of peaks appear with two maxima centered around 1460 cm^−1^ and 1580 cm^−1^ (Figure 5b). Bands in this region are associated with nucleobase vibrations, such as ring deformations and other stretching vibrations (ν(C═C), ν(C═N), ν(C–C)) (Table 1), but cannot unequivocally be assigned here. An important observation is the broad feature from 16501770 to cm^−1^ which is associated with ν(C═O) of nucleobases in DNA. This band is significantly important to provide insight into the molecular distributions in the final sensor because it is specific to ss‐DNA while the blocking agent has no absorbance in this region. Hence, the absorbance maximum of this band was imaged in the following.

IR‐mappings on Au(111)_sc_/C9t show no prominent features on a 2µm scale (Figure 5c). Significant IR‐absorbance is detected over the whole image except for the defects seen in topography and phase, which did not show IR absorbance at the absorbance maximum at 1720 cm^−1^ (Figure S4a–c, Supporting Information). The IR‐map in a smaller 500 nm scale shows slight variations in IR intensity, the associated topography and phase images are shown in Supporting Information(Figure S4d,f). To ascertain whether the topography correlates with the coincidentally recorded IR absorbance, the data point maps were analyzed statistically. Figure 5d represents a scatter plot of the recorded IR absorbance against the height value of individual pixels (1600 data points) from a selected area of the AFM‐IR images (indicated by the square in Figures 5c). The point density in this IR‐height scatter plot is represented by the colors and the plot shows a mostly circular distribution with a central cluster of the data population. This, together with the calculated Pearson correlation coefficient *r*_*PCC*_ = −0.1 with a slightly negative, but close to zero value, indicates that height and IR absorbance are not correlated. The absence of correlation indicates that the variations observed on the IR map cannot be assigned to structural differences in the SAM that are reflected in the SAM topography.

**Figure 6:**
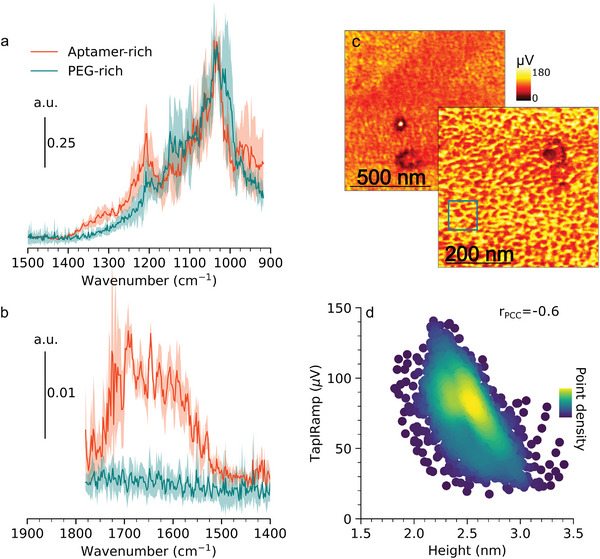
Results of TM AFM‐IR measurements on Au(111)_sc_/PEG‐C9t. Averaged nano‐IR point spectra recorded on 3–4 different positions per spectrum (a, b). Nano‐IR mapping at maximum of aptamer ν(*C* = *O*) at 1680 cm^−1^ (c) (Associated topography and phase image are given in Figure S7, Supporting Information). IR absorbance‐height scatter plot with point density represented by the color (d) created from the data in the square indicated in c.

After PEG blocking, AFM‐IR Point spectra were found to vary locally on Au(111)/PEG‐C9t with two major types of spectra (**Figure** **6**a,b). One major difference in the local spectra was found to be the appearance of a broad absorbance between 1520 and 1750cm that is assigned to the ν(C═O), ν(C═N), and ν(C═C) of ss‐DNA. Based on this observation the local spectra were grouped by the relative richness in aptamer molecules, i.e. their absorbance around 1700 cm^−1^. It is noted that grouping of the spectra by the topography of the location of spectrum recording did not yield homogenous groups, which is attributed to the fact that the observed domains are in the lateral dimensions of the resolution limit of AFM‐IR and the fact that a slight drift is always present during spectral recordings. The AFM‐IR spectra of DNA recorded on Au(111)_sc_/C9t showed three distinct peaks in the range 1400 to 1800 cm^−1^, which merge into a single broad absorption peak in the range 1520 to 1750 cm^−1^ in the aptamer‐rich spectra of Au(111)_sc_/PEG‐C9t(Figure 6b). This difference suggests a significant structural difference between the two states. The second type of spectra shows no significant absorption in this spectral region and is therefore aptamer deficient. This deficiency leads to the expectation of a relative enrichment of PEG in the second spectral type. However, in contrast to the lower wavenumber region (see discussion below), no PEG specific absorption was observed above 1400 cm^−1^ (Figure 6b). In this region, only a δ(CH_2_) (scissoring) band would be expected for PEG at about 1460 cm^−1^.^[^ ^23^ ^]^ However, this band is expected to be weak compared to the strong stretching vibrations of the more polar C═O and C═N groups measured in the aptamer‐rich spectra. Therefore, the absence of a δ(CH_2_) here may be due to insufficient sensitivity of the AFM‐IR rather than the absence of PEG.

The average aptamer‐rich and ‐deficient spectra show some common features in the spectral region below 1400 cm^−1^ but differ in their relative peak ratios (Figure 6a). In this region, the identification, and assignment of molecular vibrations to spectral features is complicated due to several overlapping bands expected from PEG and DNA. For all spectra of Au(111)_sc_/PEG‐C9t the peaks in the region from 1000 to 1250 cm^−1^ are sharper and vary from the features observed in Au(111)_sc_/C9t (cf. Figures 5a and Figure 6a). Most prominently the maximum at 1033 cm^−1^ in all spectra of Au(111)_sc_/PEG‐C9t is assigned to the symmetric stretching vibration ν_*s*_(PO_2_)^−^ of DNA showing that the aptamer is present in both sets of spectra. The ν_*s*_(PO_2_)^−^ is generally regarded to be relatively little influenced by the DNA conformation^[^ ^44^, ^48^ ^]^ and hence is used for spectral normalization, acting as reference for comparison of the two spectral sets found on Au(111)_sc_/PEG‐C9t. The comparison of the two sets of spectra reveals a relative increase in absorbance at approx. 1140 cm^−1^ which is assigned to PEGs C–O–C stretching vibration ν(C–O–C) for the aptamer deficient spectrum (Figure 6a). This observation indicates a relative enrichment of PEG molecules in the respective domains as expected for a phase‐separated mixed monolayer. The aptamer enriched spectrum on the opposite shows a relatively increased peak at ca 1210 cm^−1^ assigned to the ν_*as*_(PO_2_)^−^ of DNA.

The unambiguous assignment of aptamer‐rich and PEG‐rich domains to topographic features succeeded by means of nano‐IR mapping of the aptamer's ν(C═O) at 1680 cm^−1^ (Figure 6c, associated topography and phase images; Figure S7, Supporting Information). The intensity in the IR maps varies significantly on the surface and exhibits similar nano‐domain patterns as the topographic images. This visual assessment is supported by the height‐IR scatter plot which shows a non‐unimodal and elongated distribution of data points with a negative slope and yields a correlation coefficient of ‐0.6. This result evidences an anti‐correlation between IR absorbance at 1680 cm^−1^ and the height or in other words that elevated domains show a reduced IR absorbance. Taken together, nano‐IR mapping confirms that the sensing layer is phase separated into an aptamer‐rich phase and a PEG‐rich phase while the latter is, as shown by AFM topography, elevated up to 1.5nm above the aptamer‐rich domains. Spontaneous nanoscale phase separation is a well known general phenomenon in binary mixed SAMs of alkanethiols that is governed by the interplay of mixing entropy and intermolecular interactions.^[^ ^49^, ^50^, ^51^, ^52^ ^]^ However, for SAMs of (bio‐)macromolecules phase separation is much less reported. Recently, phase separation has been shown to occur in an electrochemical malaria sensor and degrade the efficiency of analyte binding for phase separated mixed ss‐DNA/PEG monolayer.^[^ ^17^ ^]^ On the contrary, the fabrication method employed here follows a protocol that was optimized for sensor sensitivity.^[^ ^13^ ^]^ Apparently, the phase separation does not have a strongly detrimental effect on the sensor performance in this case. Remarkably, the C9t aptamer assembles in domains that are lower in thickness compared to the domains containing predominantly the shorter PEG molecules. Hence, it is assumed that intermolecular interactions such as base pair stacking and hairpin formations influence the conformation of the aptamer‐rich phase and are possible driving forces for the observed phase separation.

### 2.4 Observation of Single Molecule Analyte Binding

**Figure 7:**
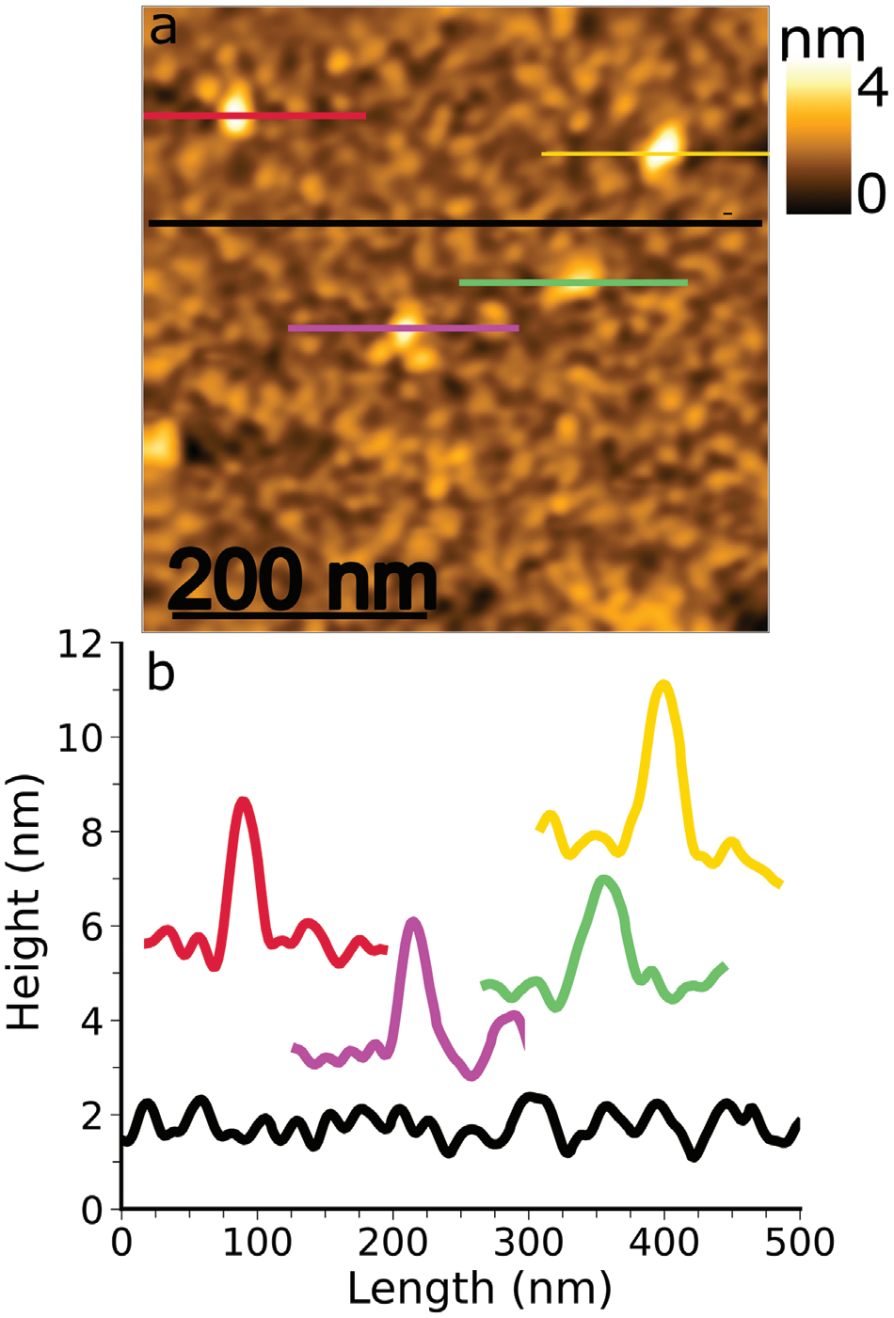
Results of TM AFM of Au(111)_sc_/(PEG‐C9t)+ S protein topography image (a) and line profiles (b). The line profiles are in corresponding colors to the lines in the topography image.

After the incubation of the Au(111)_sc_/PEG‐C9t sample in the spike protein solution, elevated globular features were observed in AFM topography that were absent before (**Figure** **7**a). The features' shapes are dominated by the AFM tip shape, and they protrude approx. 3 to 4nm from the receptor layer from their line profiles (Figure 7b). The line profile on the uniform receptor layer (Figure 7b) illustrates height variations of 1nm similar to the phase seperated Au(111)_sc_/PEG‐C9t (Figure 2c). The comparison of the AFM topographies before and after protein incubation shows that the appearance of the elevated features resulted from the protein incubation. Hence, the features are assigned S proteins and further evidence for this assignment is provided by the nano‐IR results.

**Figure 8:**
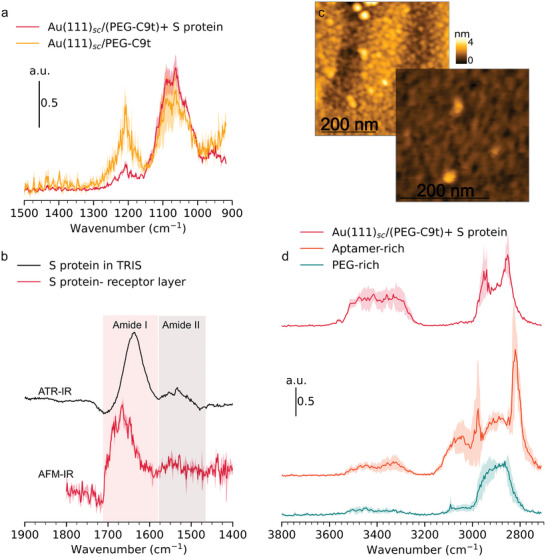
Results of TM AFM‐IR measurements on Au(111)_sc_/(PEG‐C9t)+ S protein. Averaged nano‐IR point spectra recorded on proteins and receptor layer (a). Comparison of solution spectrum of S protein recorded by ATR‐IR with difference spectrum S proteins minus receptor layer recorded by AFM‐IR(b). Topography image of proteins complex on receptor layer (c). Averaged nano‐IR point spectra recorded on proteins and nano domains in the receptor layer (d).

Nano‐IR point spectroscopy on protein features was used to study their chemistry and nature. Point spectra on several features and on random positions next to them on the receptor layer were recorded and averaged (**Figure** **8**a–d). In the range from 1000 to 1350 cm^−1^, the spectra (normalized at 1030 cm^−1^) show two prominent bands assigned to asymmetric and symmetric phosphate stretching vibrations at approx. 1210 cm^−1^ and approx. 1060 cm^−1^

Characteristic spectral features that can be assigned specifically to the protein lie in the range from 1520 to 1710 cm^−1^ (cf. Table 1). Compared to the spectra of the receptor layer, the protein spectra show a slight increase in intensity from 1520 to 1560 cm^−1^ and a notably strong increase in intensity from 1600 to 1710 cm^−1^ (Figure S8a, Supporting Information). They are assigned to amide II and amide I bands of the S proteins bound to the sensor. The difference spectrum, calculated by subtracting the averaged receptor layer spectra from the averaged protein spectra, clearly features both amide bands (Figure 8b). The spectra of the receptor layer in the same region show a broad lower intense feature that resembles the absorption observed in spectra of aptamer‐rich domains in Au(111)/PEG‐C9t (Figure S8a and Figure 6b).

It is interesting to compare the structural properties of the sensor bound protein to the solution structure. To that end, an ATR‐IR spectrum of the protein in solution was recorded. The comparison with the nano‐IR difference spectrum of the adsorbed proteins shows that the latter is significantly shifted to higher frequencies (Figure 8b). The S proteins in solution have the Amide I maximum at approx. 1635 cm^−1^ while the adsorbed protein is centered clearly above 1650 cm^−1^ (exact value 1665). This shift indicates that the secondary structure of the surface‐bound S protein is significantly altered as compared to the solution structure. This goes in hand with the observed relatively low height of the protein in the AFM topography compared to its solution structure. The S protein is known to be a trimer with major axes dimensions of approx. 10nm and 15nm.^[^ ^53^ ^]^ The found heights of approx. 4nm imply that either the protein immerses into the receptor layer or that a significant structural rearrangement of the protein takes place upon binding as indicated by the nano‐IR results. Since solid/liquid interfaces pose a significantly different chemical environment compared to bulk solvents, protein restructuring is an often observed phenomenon upon adsorption^[^ ^54^ ^]^ and is evidently occurring here during analyte binding.

Next, nano‐IR spectra with a different laser covering the higher wavenumber range from 2700 to 3800 cm^−1^ were recorded on Au(111)_sc_/PEG‐C9t incubated with S protein (Figure 8d). Broad and strong bands were observed in the range from 2800 to 3000 cm^−1^ for all spectra (Table 1). The peaks in this region can be assigned to asymmetric and symmetric C–H stretching vibrations of aliphatic methylene and methyl groups as they exist in all components in deoxyribose of DNA, in PEG, and protein side chains. Notably, the spectra for the PEG‐rich phase feature a broad peak with a maximum at approx. 2860 cm^−1^ with a high‐frequency shoulder at approx. 2930 cm^−1^. Such a ν(CH) spectrum is typical for liquid PEG^[^ ^55^, ^56^, ^57^ ^]^ and hence clearly shows that PEG chains are amorphous, in line with the results of the layer thickness determination.

A feature from 3200 to 3600 cm^−1^ associated with ν(OH)‐water and/or ν(NH) appears in all spectra with mild intensity for aptamer‐rich domains and very strong intensity in protein spectra. This could be associated with the higher contribution of these groups in the molecular structure. Also, water may remain among molecules with hydrophilic moieties, forming strong hydrogen bonds within the complex and not being removed by the drying of the sample. Imaging of the surface‐bound S protein succeeded at the amid I maximum at 1660 cm^−1^ as well as with the other laser at the ν(OH) at 3350 cm^−1^ (Figure S8b,c).

A significant broad band in the 3000 to 3160 cm^−1^ range with a maximum at approx. 3050 cm^−1^ is found in the spectrum of aptamer‐rich domains (Figure 8d). Bands in this spectral region are specific for C–H stretching vibrations of aromatic rings (ν(C_*ar*_‐H) and this band is found very weakly in the spectra of the protein and the PEG‐rich phase. Clearly, a relatively high fraction of aromatic C_*ar*_‐H is prevalent in the nucleobases of ss‐DNA, while the abundance is much less in natural amino acids and hence in the protein as well. Also, it is expected that ss‐DNA binds the target protein and thus the appearance of the ν(C_*ar*_‐H) band in the protein spectrum is not surprising. However, no C_*ar*_‐H exists in the PEG molecule. The occurrence of the ν(C_*ar*_‐H) band in the spectrum of the PEG rich phase is similar to the occurrence of the ν_*s*_(PO_2_)^−^ in this phase. One might attribute this to an intermixing of aptamer in the PEG, however, it must be remembered that the resolution of AFM‐IR is limited by the tip size.^[^ ^35^ ^]^ The lateral resolution for the measurements reported here, was determined to be about 15 to 20nm (Supporting Information, Figure S7g), which is in the range of the lateral domain size observed here. This means that it cannot be unambiguously concluded whether the PEG‐rich phase consists exclusively of PEG or has the aptamer intermixed. Taken together nano‐IR spectroscopy in the higher wavenumber range supports the conclusion of a phase separated receptor layer and the observation of analyte binding on the single molecule level.

## 3 Conclusion

In this study, nanoscopic details of the structural changes occurring during the fabrication of a functional aptamer‐based biosensor and upon analyte binding are reported. In a first step, the ss‐DNA immobilization creates a homogenous and relatively thin layer of unordered DNA molecules. Next, additional PEG surface tethering, thickens the layer and hence causes the DNA chains to rise up accompanied by a nanoscale phase separation. Nano‐IR spectroscopy and imaging provided unique chemical information on the adlayer composition and proved that the domains consist of an aptamer‐rich and a PEG‐rich domain of higher thickness with the PEG chains being in an amorphous conformation. Furthermore, single molecule nano‐IR‐spectra of individual sensor‐bound spike proteins were measured, which indicates a significant change in the analyte's secondary structure upon the formation of the aptamer‐protein complex. The factors governing such nano‐structural properties and their influence on analyte binding should be the subject of future systematic studies with the aim to gain a deep understanding of the (physico‐)chemical processes at the aptasensor surface on the nanoscale. Nano‐IR investigations have provided insights into the chemical processes of biosensor's receptor layers, thus advancing the understanding of interfacial binding events on the nanoscale and even the level of individual molecules.

## 4 Experimental Section

### Materials

Buffers were prepared in ultra‐pure water with 18.2 MΩ cm^−1^ resistivity (ELGA LabWater). Tris‐HCl buffer contained 25mM tris(hydroxymethyl)aminomethane, 8mM HCl and 100mM NaCl at pH 7.4 yielding 150mM ionic strength. High salt phosphate buffered saline (hs‐PBS Mg^2 +^) contained 1mM MgCl_2_, 10.0mM PO4^3 −^ and 1M NaCl at pH 7.0‐7.2, yielding 1.14M ionic strength. Truncated ss‐DNA aptamer C9t (FRIZ BIOCHEM GmbH, Germany) with 40 bases as described in ref. [13] was used. The C9t aptamer was purchased with modification on its 5'‐end with 2 groups of dithiol phosphoramidite (DTPA). Tris(2‐carboxyethyl) phosphine 10mM (TCEP, Sigma‐Aldrich, Munich, Germany) was used to break the disulfide bond of the aptamers' DTPA. The PEG‐blocking molecule poly(ethylene glycol) methyl ether thiol 2000 (Sigma‐Aldrich, Germany) was used with 5mg/mL concentration dissolved in the tris‐HCl buffer. The full‐length Spike (S) protein of the Omicron variant of SARS‐CoV‐2 virus with a molar mass of 142114g mol^−1^ (2019‐nCoV, Sino Biological, Germany) was used as the target analyte dissolved in tris‐HCl buffer. Two different substrates, namely gold single crystal with predominantly (111) orientation (Mateck GmbH, Germany) and poly‐crystalline evaporated gold (250nm) on glass (Arrandee metal GmbH + Co. KG, Germany) were used for AFM‐IR and IRRAS measurements respectively. 1‐Hexadecane‐d33‐thiol, HDTD_33_ (CDN ISOTOPES GmbH, Germany) was used for IRRAS background measurements. 99.9% ethanol (Merck, Germany) was used for dilution and rinsing.

### Sample Preparation

The initial step was the immobilization of aptamer receptors on an ultra‐flat Au(111) surface. The Au(111) terraces were achieved by flame annealing of the polycrystalline Au(111) or single crystal Au(111)_sc_ substrate in a hydrogen flame for ∼3 to 10min followed by cooling in a nitrogen stream. For the preparation of the background sample, the Au(111) was immersed in 0.1mM HDTD_33_ in argon‐saturated ethanol overnight, followed by subsequent rinsing steps with absolute ethanol, ultra‐pure water, and drying in a nitrogen stream. The sulfide groups of multi‐thiol functionalized C9t aptamer were reduced with TCEP (10mM), with a volume ratio of 1:3 (aptamer:TCEP) and further diluted in hs‐PBS Mg^2 +^ to reach 0.1µ M and 0.5µ M of aptamer for macroscopic and nanoscopic measurements, respectively. Activated aptamers were immobilized as receptors on gold substrates (Au(111)/C9t) by incubation for 2 h and subsequent rinsing with hs‐PBS Mg^2 +^, tris‐HCl, and ultra‐pure water to remove non‐bound molecules and salts and a final drying under a nitrogen stream. For the blocking step, the aptamer‐modified sensor was immersed in PEG 2000 (0.5µ M) dissolved in tris‐HCl for 1 hour (Au(111)/PEG‐C9t), followed by rinsing with tris‐HCl, ultra‐pure water, and dried under a nitrogen stream. For the protein binding experiment (Au(111)/(PEG‐C9t)+ S protein), the sensor was immersed in 30ng/mL of S protein in tris‐HCl buffer for 45 min for AFM‐IR measurements and in 0.015ng/mL of S protein in the same buffer overnight for IRRAS measurements. The sample was rinsed gently with tris‐HCl, ultra‐pure water, and was dried by nitrogen stream. A higher protein concentration was chosen for the AFM‐IR experiments to maximize the amount of retained analyte after rinsing and drying to facilitate the detection of analyte by AFM.

### Infrared‐Reflection‐Absorption Spectroscopy, IRRAS

IRRA spectra after each fabrication step were recorded at p‐polarization using an external reflection unit (Seagull, Harrick Scientific Products, Inc., New York) in a nitrogen environment at an incident angle of 72° in a Bio‐Rad FTS 3000 Excalibur Series FT‐IR spectrometer equipped with a mercury‐cadmium‐telluride (MCT) detector and KBr beam splitter. Each spectrum was recorded with an accumulation of 256 scans using an aperture of 0.25 cm^−1^ and a resolution of 4 cm^−1^ from 900 to 4000 cm^−1^. A flame annealed Au(111) was used a background and water subtraction. The spectra are averaged but are not normalized.

### Attenuated Total Reflection IR Spectroscopy, ATR‐IR

ATR‐IR spectra were recorded on a Bio‐Rad Excalibur spectrometer in wavenumber region of 800 to 4000 cm^−1^ equipped with a mercury‐cadmium‐telluride (MCT) detector. A multi reflection ATR unit (Spectra‐Tech, Inc.) was used. Double side polished, N‐type doped Ge(100) crystals (Nano Quartz Wafer GmbH, Germany) of size 52mm × 20mm × 0.5mm were used as internal reflection elements. The Ge substrates were polished into a trapezoidal shape giving an incident angle of 60°. IR spectra were recorded in p‐polarization with 256 scans and spectral resolution of 4 cm^−1^. In a home build liquid cell mounted on the Ge crystal, first several IR spectra of the 650µ L tris‐HCl buffer were collected as background and for use of the water vapor subtraction. The buffer was removed and IR spectra in 650µ L of S protein (0.015ng/mL, 0.015µ M) in tris‐HCl were recorded. Spectra were corrected for water vapor by factored subtraction of a respective spectrum and averaged.

### Spectroscopic Ellipsometry, SE

A spectroscopic ellipsometer SE 800 (SENTECH instrument GmbH, Germany) with SpectraRay/4 software was employed for the measurement and data analysis. Spectra of the ellipsometric Δ angle were recorded at an incident angle of 80° for Au(111) and after each modification step and analysed in the range where the SAM was found to be transparent: 600 to 825nm. Assuming isotropic and homogeneous layers, an optical model of 3‐layers (ambient | layer | substrate) was defined. The substrate optical constants were determined by inversion of the experimental data measured on similar Au(111) substrates. For the SAM layers a wavelength independent refractive index of *n* = 1.43 was used, and for the ambient air *n* = 1. The spot size diameter on the sample was on the order of 2 mm^2^ . The resulting thicknesses are averaged over 9 positions on two or three samples. Since the commercially received Au substrates used for SE measurements were not completely opaque, the determined optical constants of Au(111) deviated from sample to sample as well as from the ideal reflectivity in bulk gold. The error in thickness determination caused by these deviations was determined by applying the same 3‐layer model to several substrate measurements.

### Nano‐IR Spectroscopy, AFM‐IR

For AFM‐IR, a Bruker nano‐IR 3 (Bruker Corporation, USA) was employed. Measurements were taken after each sensor fabrication step. Different tunable Mid‐IR laser sources were used, a Quantum Cascade Lasers (QCL, MIRcat, DRS Daylight Solutions) to cover the 905 to 1970 cm^−1^ range, consisting of four chips with narrower spectral ranges (with transition steps at 1204.7 cm^−1^, 1432.6 cm^−1^ and 1709.3 cm^−1^). Also, a fast Optical parametric oscillator (OPO, firefly laser, M Squared Lasers Inc.) to cover the 2700 to 3800 cm^−1^ range was used. Tapping mode AFM‐IR was performed to preserve the surface from damage caused by strong tip‐sample interactions. A gold‐coated silicon probe tip with a nominal fundamental resonance frequency of 75kHz± 15kHz (Tapping Mode NIR2 Probes, PR‐EX‐TnIR‐A‐10, ANASYS INSTRUMENTS) was employed to measure topography, phase, IR‐mapping, and point spectra. The topography was recorded at the constant amplitude oscillation at the fundamental free resonance between 69 to 76kHz. The repetition rate of laser pulses was set to the difference between two eigenmodes of cantilever free oscillations, 370 to 388kHz for the QCL lasers and 190 to 191kHz for OPO laser. The pulse frequency was tracked and controlled during IR‐mapping measurements by enabling a phase‐locked loop (pll) to correct pulse tuning for variations in the contact frequency of the cantilever during the scan. Pulse widths of 100 to 480ns were chosen depending on the sample sensitivity and measurement conditions. For measuring protein single molecules an off‐resonance tuning scheme was employed.^[^ ^37^ ^]^ The laser power was optimized for obtaining a good signal but sometimes had to be lowered in cases of very high IR amplitudes due to a loss of linearity of the photothermal signal as reported earlier.^[^ ^37^ ^]^ The laser power were between 3.8 to 64% of the maximum peak power for the four modules of QCL, i.e. 0.2 to 9mW and for the OPO laser from 0.1 to 1.5mW (2 to 6%). By default, the spectral intensities were corrected for the laser output power. The images were recorded in scan sizes of 0.3 to 3µ m with 256 × 256 pixels and scan rates of 0.5 to 1Hz. The presented images were smoothed by Gaussian filter to remove the background noise, the roughness analysis was performed on the unfiltered images. The nano‐IR point spectra were corrected using the step discontinuity module in Analysis Studio software to remove artifacts caused by the QCL chip transitions. The spectra were normalized at a common and maximum peak and averaged. The normalization was performed to exclude the influence of tip‐surface specifications using different tips or encountering various surface roughness or laser power fluctuations. The spectra on Au(111)_sc_/C9t were normalized at approx. 1030 cm^−1^ assigned to ν_*s*_(PO_2_)‐. The spectra on Au(111)_sc_/PEG‐C9t, in the range from 905 to 1500 cm^−1^ were also normalized at ν_*s*_(PO_2_)‐ to visualize the impact of PEG tethering regardless of molecular orientation change in the spectra. The spectra were normalized at 954 to cm^−1^ in the range from 1400a to 1900 as a common band for an optimum signal‐to‐noise ratio. In the higher wavenumber region, the spectra were normalized at 2865 cm^−1^ assigned to ν(CH) vibration.

## Conflict of Interest

The authors declare no conflict of interest.

## Supporting information

Supporting Information

## Data Availability

The data that support the findings of this study are available from the corresponding author upon reasonable request.
