# Supplementary material for: Nano‐Phase Separation and Analyte Binding in Aptasensors Investigated by Nano‐IR Spectroscopy
Source: Small. 2025 Jan 19;21(19):2409369. doi: 10.1002/smll.202409369 (PMC12067171; doi:10.1002/smll.202409369)
Supplement: Supplementary file 1 — Supporting Information [file SMLL-21-2409369-s001.pdf]

NANO · MICRO
small

Supporting Information

for *Small*, DOI 10.1002/smll.202409369

Nano-Phase Separation and Analyte Binding in Aptasensors Investigated by Nano-IR Spectroscopy

*Nafiseh Samiseresht, Gabriela Figueroa Miranda, Ankita Das, Kevin Graef, Dirk Mayer and Martin Rabe**

Supporting Information for: Nano-phase separation and analyte binding in aptasensors investigated by nano-IR spectroscopy

Nafiseh Samiseresht, Gabriela Figueroa Miranda, Ankita Das, Kevin Graef, Dirk Mayer, and Martin Rabe*

E-mail: m.rabe@mpi-susmat.de

Contents

Supporting discussion 1 **2**

Supporting discussion 2 **5**

Supporting Figures **8**

Supporting discussion 1: Granular features observed in AFM topography

TM-AFM topographies on $Au(111)_{sc}$ after flame annealing show the appearance of protrusions on ultra-flat terraces of $Au(111)_{sc}$ (figure S1). By means of a sharp tip the herringbone reconstruction was observed in the phase image (figure S1d), which indicate that a clean Au(111) interface is present. The protrusion features were also identified in scanning electron microscope (SEM) images (figure S2). The inverted contrast in the image recorded with the electron backsattering detection (BSD, figure S2a) show that the features are composed of elements lighter than the surrounding gold. Additional energy-dispersive X-ray (EDX) analysis of the protrusions was performed (figure S3). The EDX spectra are dominated by peaks that can be assigned to Au (M: 2.1 keV), while small features can be found also for Al (K_α: 1.5 keV), Si (K_α: 1.7 keV) and O (K_α: 0.5 keV). These peaks are assigned to remaining polishing agents which are typically AlOx or SiC slurrys. The protrusions that remained after surface modification with aptamer show a height of 2 nm and 6 nm in the line scan (figure S4a). Nano-IR mapping on $Au(111)_{sc}$/C9t at 1720 cm^{-1} and other wavenumbers reveals that they are of inorganic nature with zero IR absorbance (figure S4b). Hence, no SAM formation occurred on the residuals and their presence had no discernible influence on the SAM morphology on the gold terraces.

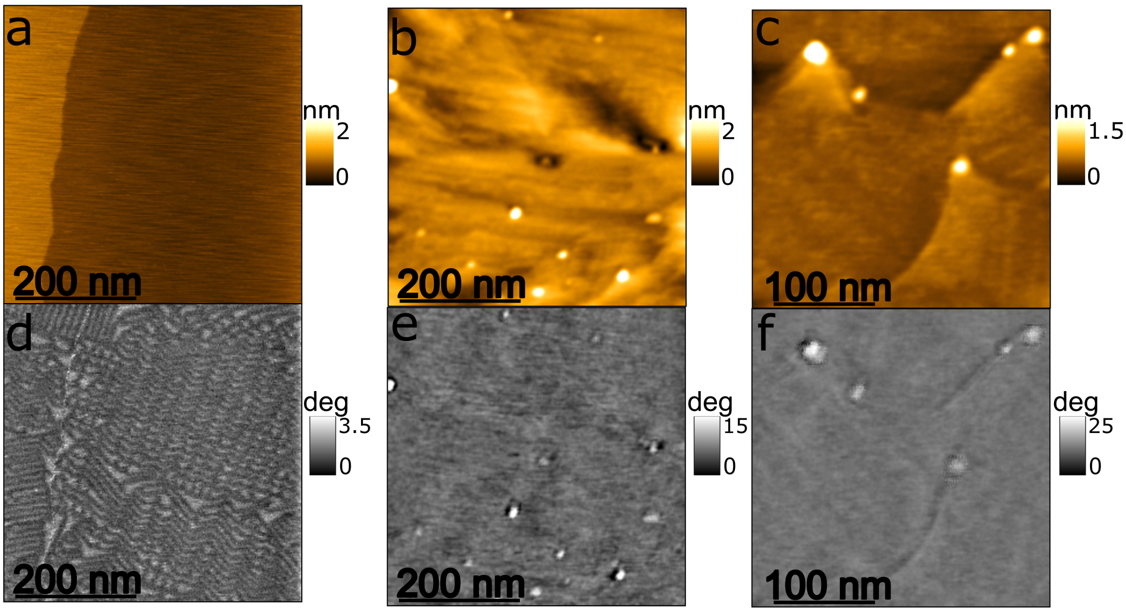

Figure S1: Results of TM-AFM topography on Au(111)$_{sc}$ after flame annealing. The topography (a, b, c) and phase (d, e, f) of small and large scan areas. (a, d) are recorded by a sharp silicon tip. Large terraces of Au(111) with herringbone reconstruction are resolved. (b, c, e, f) Show large residuals on Au(111) surface.

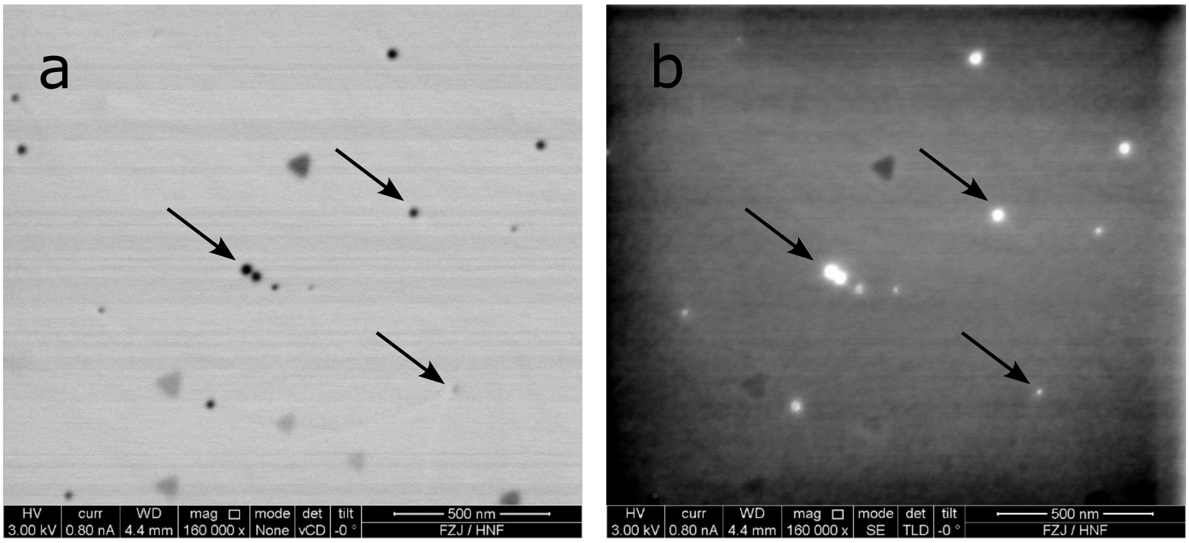

Figure S2: SEM image of Au(111) surface after flame annealing recorded by a backscatter detector (a). Arrows mark spherical objects that are responsible for step pinning, which appear dark, suggesting a lower atomic number than the surrounding Au atoms. SEM image of the Au (111) surface at the same location recorded with a secondary electron detector (b). The arrows indicate the same objects which appear bright due to their elevated topography.

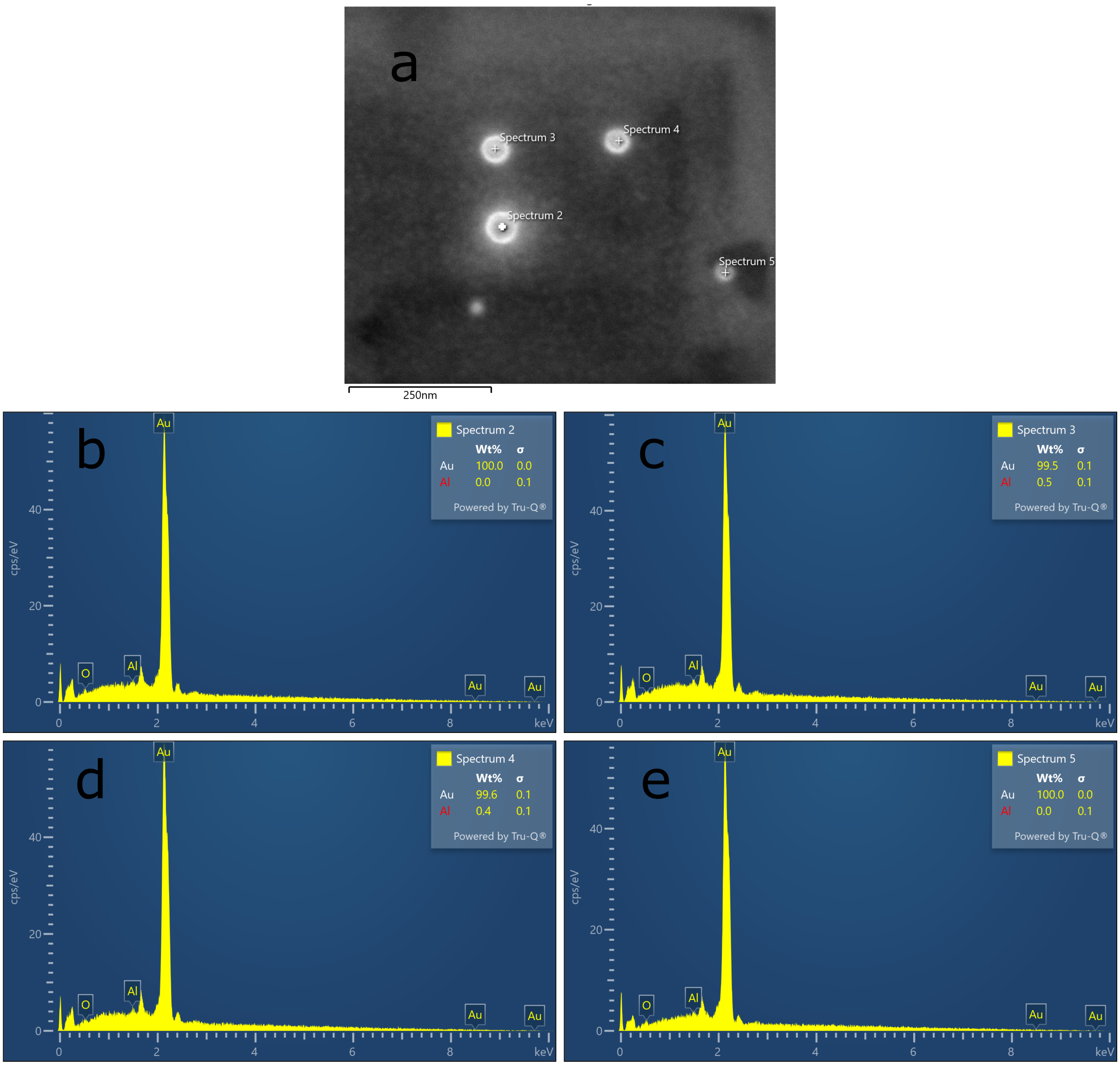

Figure S3: SEM image recorded at a different location on the same sample as Fig. 2 (a). The crosses indicate the locations where EDX spectra were recorded. The corresponding EDX spectra (b-e) with spectra 2, 3, 4, 5 assigned to the corresponding locations in the SEM image in a.

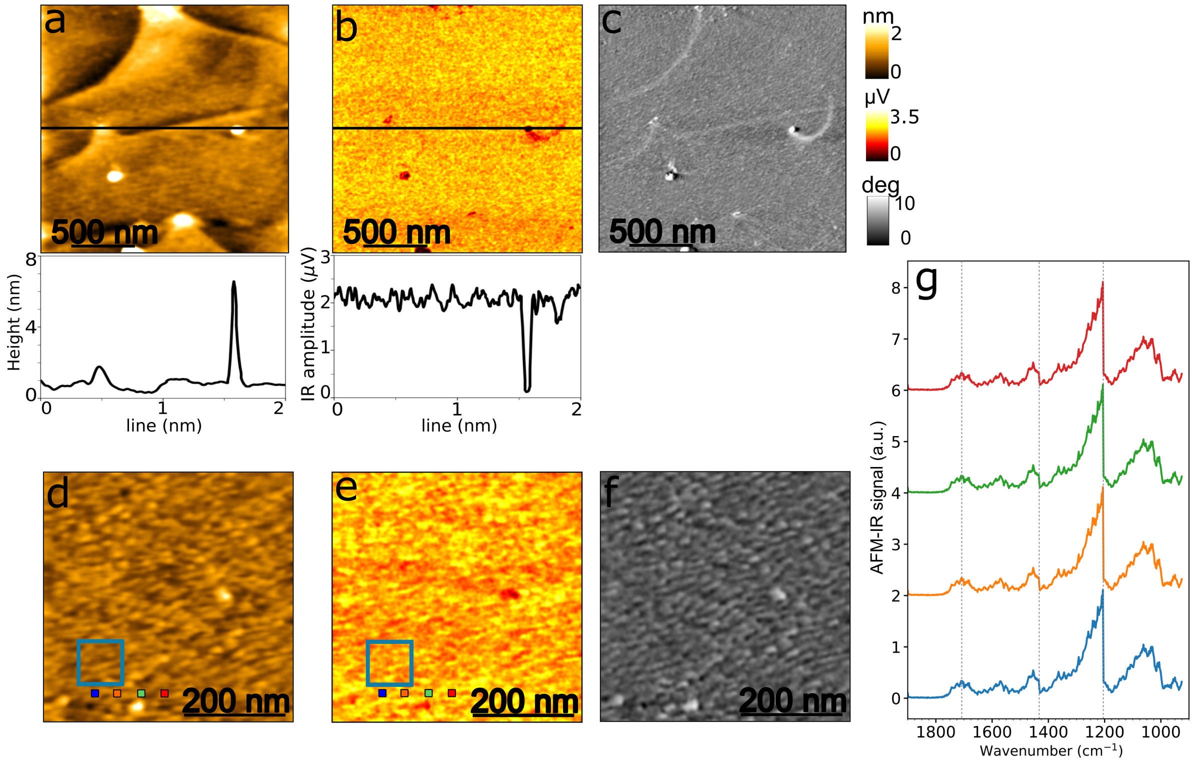

Figure S4: Results of TM-AFM topography, nano-IR mapping at 1720 cm^{-1} and phase on $Au(111)_{sc}/C9t$, in large scan area (a-c) and small scan area (d-f). The Au adatoms and triangular reconstruction of $Au(111)_{sc}$ are removed, but large protrusions are still on the surface with the height of 2 and 6 nm in the line scan (a). Nano-IR mappings show minimum intensity on the protrusion of 6 nm size (b). The protrusions show stronger variation in stiffness and adhesion compared to the rest of the surface (c). The squares are indicative of the area used for the height-IR correlation plot (d, e) phase image (f). The nano-IR spectra (g) were recorded at the positions indicated on a snippet of the height and IR maps (purple rectangle in e, d), with a color code used for identification. The spectra are normalized at 1030 cm^{-1} The dashed lines are QCL transition steps.

Supporting discussion 2: Details on spectroscopic ellipsometry data analysis and modeling

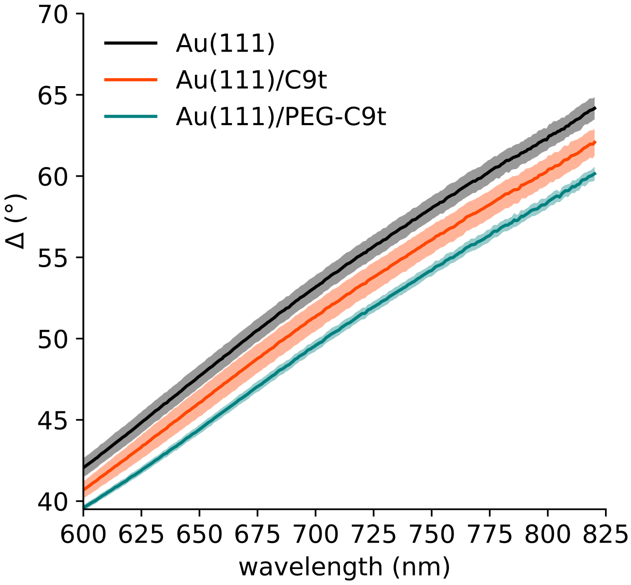

Figure S5: Values of Δ measured by spectroscopic ellipsometry. Shaded areas represent standard deviations.

Table S1: Results of Welch's t-test applied for Δ(650 nm).

	t-statistic	deg. freedom	$p_{two-tail}$
Au(111) - Au(111)/C9t	3.96925	14.37	0.00133
Au(111)/C9t - Au(111)/PEG-C9t	5.17368	9.76	0.00045
Au(111)/PEG-C9t - Au(111)/(PEG-C9t)+ S protein	-0.01819	14.07	0.98574

Figure S5 shows the measured Δ values and their standard deviations for three samples with three measurements per sample for Au(111) , Au(111)/C9t , and Au(111)/PEG-C9t . The results of Au(111)/(PEG-C9t)+ S protein are omitted for clarity because its curve largely overlapped with Au(111)/PEG-C9t . It is noted that the standard deviations of the raw Δ values imply significant differences between the optical thicknesses ($n \times d$) of the different systems. To test for statistically significant differences, Welch's t-test was performed for Δ values at 650 nm (table 1). The low p values below 0.5 % for the comparison of the Δ values of Au(111) vs. Au(111)/C9t and Au(111)/C9t vs. Au(111)/PEG-C9t indicate that the optical thickness is significantly increased during the two sensor fabrication steps. The higher uncertainties in the absolute thicknesses reported in the main part (figure 3) arise

from the combination of the errors of the substrate and the respective samples.

The determination of the SAM thickness is done under specific simplified assumptions, which are:

1. a constant refractive index of $n = 1.43$[1] without dispersion describes the optical properties of the SAMs in Au(111)/C9t and Au(111)/PEG-C9t
2. the SAMs are optically homogenous film
3. a three-layer model (substrate | SAM | air) describes the complete system

However, as shown by AFM in the main part of the study, the assumption of a homogenous film is not correct in the case of Au(111)/PEG-C9t . Hence, the obtained thickness values represent the values that would give a similar optical response in the 3-layer model with the above assumptions.

Also, it must be noted that for thin transparent films as treated here, spectroscopic ellipsometry can only determine the optical thickness $n \times d$. Hence, n and d are highly correlated, and precise knowledge of one of the two dimensions is required to precisely determine the other.[2] According to the Clausius-Mosotti equation, the film refractive index n depends on the film density and the polarizability. Hence, it is very likely that PEG blocking increases n as well as d, as for instance observed upon blocking of ss-DNA film with short alkanethiols.[3] Values of n between 1.45 to 1.5 are typically used for dense and well-ordered films of aliphatic thiols[4,5] and found in dense DNA films.[6,7] Pinto et al. found n between 1.45 to 1.43 in the vis-NIR range above 650 nm for a dense ss-DNA film prepared at high ionic strength, with an independently determined thickness of approx. 3 nm.[1] The authors also showed the dependency of ss-DNA density and thickness on ionic strength. The values found here for Au(111)/C9t , are very similar to that result. A less dense ss-DNA layer was characterized by n about 1.4.[3]

The statistical analysis of differences in $\Delta(650\,\text{nm})$ showed that the optical thickness is increased upon PEG blocking and it is considered likely that this is due to both an increase in n and d. However, because the actual value and increase upon blocking in n is unknown the numerical increase of d by approx. 3.4 nm can be understood as a maximum limit of the thickness increase. To estimate the influence of variations in n on the calculated thickness,

different extreme values for n were tested in a fitting routine. It was found that assuming a rather low $n = 1.36$ for the layer on Au(111)/C9t would give an average thickness of 3.3 nm while for Au(111)/PEG-C9t a rather high $n = 1.5$ would result in $d = 5.7$ nm. Or in other words, assuming a strong increase of n from 1.36 to 1.5 upon PEG blocking would result in a slightly lower increase in the mean thickness from 3.3 nm on Au(111)/C9t to 5.7 nm on Au(111)/PEG-C9t . Due to the simplifications imposed on n, usage of a more precise 4-layer model as repeatedly described in literatures (for instance[1,3]) is not considered useful here, since it will not improve the significance of the obtained thickness values.

Supporting Figures

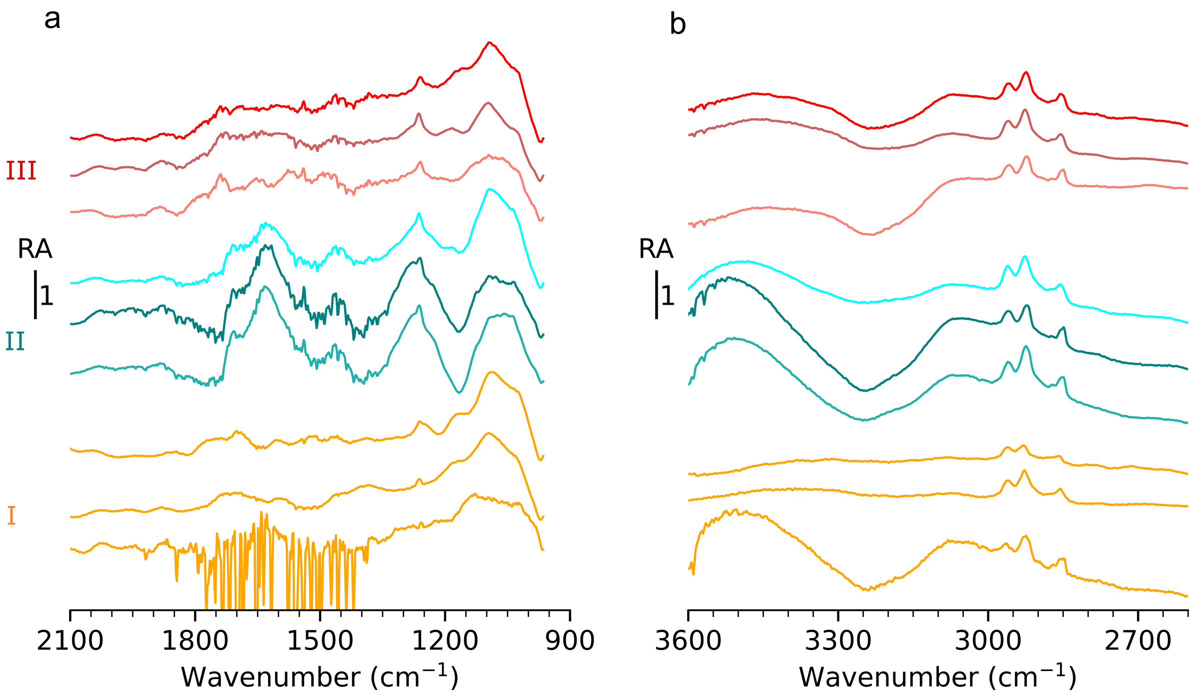

Figure S6: IRRA spectra in the low (a) and high wavenumber range (b). Three samples after; step (I) on Au(111)/C9t , step (II) on Au(111)/PEG-C9t , and step (III) on the respective sensor bond with S protein. The ν $(PO_2)^-$ in (a) and $\nu(CH_2CH_3)$ bands in (b) are observed.

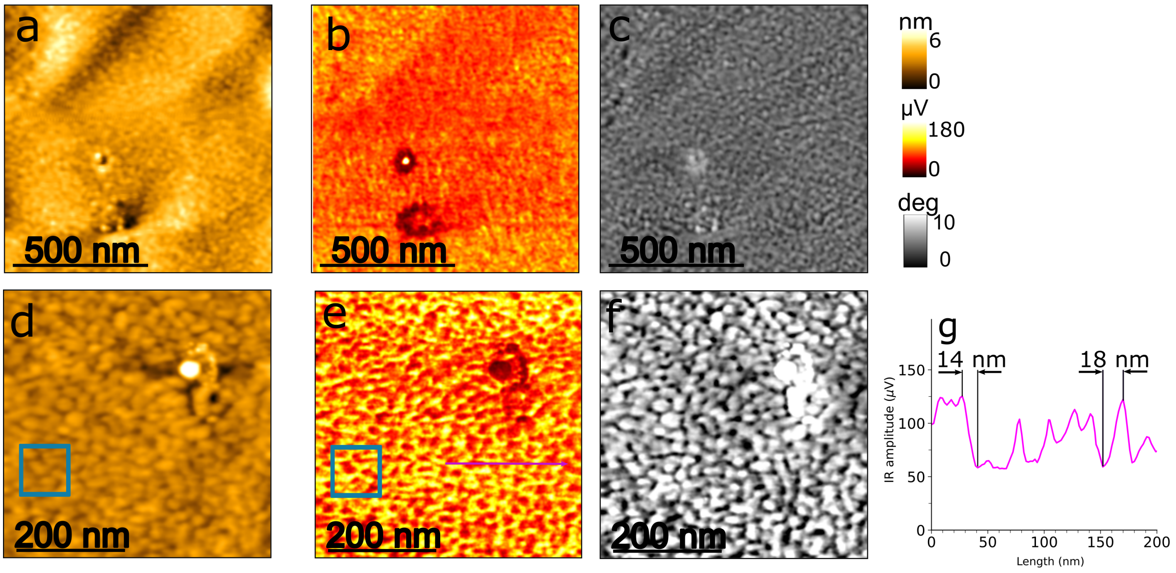

Figure S7: Results of TM-AFM topography, nano-IR mapping at 1680 cm^{-1}, and phase on Au(111)/PEG-C9t . In large (a-c) and small scan areas (d-f). The squares are indicative of the area used for the height-IR correlation plot. A spatial resolution better than 20 nm is illustrated in (g) for the data along the line in the IR-map in (e).

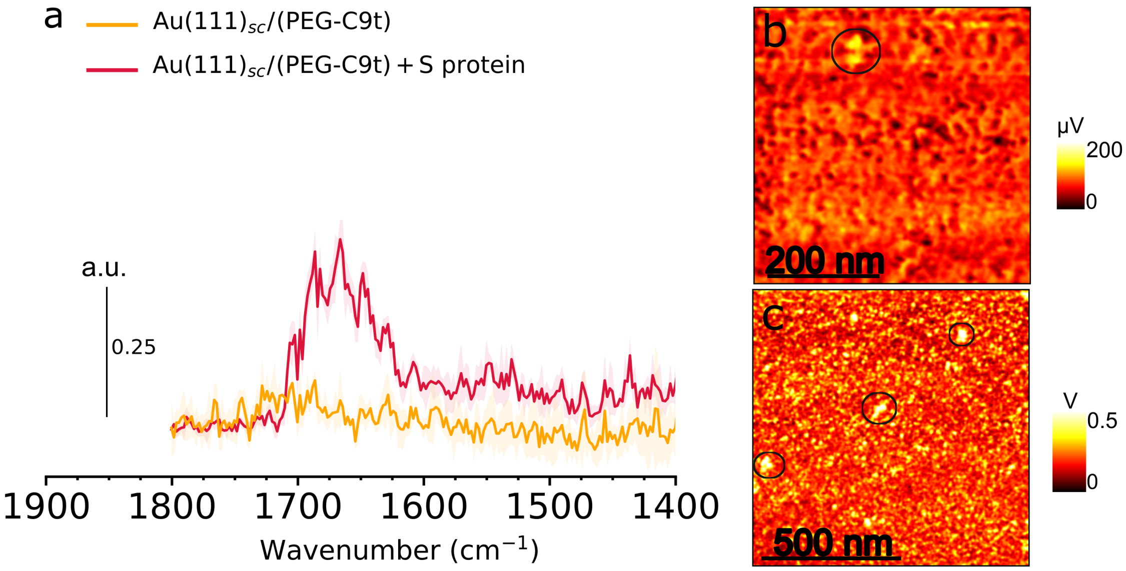

Figure S8: Results of TM-AFM IR. Averaged nano-IR point spectra on the proteins and the positions aside from proteins (measured on 4 proteins, normalized at 954 cm^{-1}) (a). Nano-IR mappings at 1660 cm^{-1} (b) and 3350 cm^{-1} (c) with high intensity on the protein-bound complexes (in the circles).

References

1. Pinto, G.; Canepa, P.; Canale, C.; Canepa, M.; Cavalleri, O. Morphological and Mechanical Characterization of DNA SAMs Combining Nanolithography with AFM and Optical Methods. *Materials* **2020**, *13*, 2888.

2. Arwin, H. *Handbook of Ellipsometry*; William Andrew Publishing: Norwich, NY, 2005.

3. Pinto, G.; Parisse, P.; Solano, I.; Canepa, P.; Canepa, M.; Casalis, L.; Cavalleri, O. Functionalizing gold with single strand DNA: novel insight into optical properties via combined spectroscopic ellipsometry and nanolithography measurements. *Soft Matter* **2019**, *15*, 2463–2468.

4. Nuzzo, R. G.; Allara, D. L. Adsorption of bifunctional organic disulfides on gold surfaces. *J. Am. Chem. Soc.* **1983**, *105*, 4481–4483.

5. Bain, C. D.; Troughton, E. B.; Tao, Y. T.; Evall, J.; Whitesides, G. M.; Nuzzo, R. G. Formation of monolayer films by the spontaneous assembly of organic thiols from solution onto gold. *J. Am. Chem. Soc.* **1989**, *111*, 321–335.

6. Elhadj, S.; Singh, G.; Saraf, R. F. Optical Properties of an Immobilized DNA Monolayer from 255 to 700 nm. *Langmuir* **2004**, *20*, 5539–5543.

7. Legay, G.; Markey, L.; Meunier-Prest, R.; Finot, E. Measurements of thickness dispersion in biolayers by scanning force microscopy and comparison with spectroscopic ellipsometry analysis. *Ultramicroscopy* **2007**, *107*, 1111–1117.
